# The acclimatization strategies of kidney vetch (*Anthyllis vulneraria* L.) to Pb toxicity

**DOI:** 10.1007/s11356-018-2197-6

**Published:** 2018-05-07

**Authors:** Barbara Piwowarczyk, Krzysztof Tokarz, Ewa Muszyńska, Wojciech Makowski, Roman Jędrzejczyk, Zbigniew Gajewski, Ewa Hanus-Fajerska

**Affiliations:** 1 Unit of Botany and Plant Physiology, Institute of Plant Biology and Biotechnology, Faculty of Biotechnology and Horticulture, University of Agriculture in Krakow, Al. 29 Listopada 54, 31-425 Kraków, Poland; 2 Department of Botany, Faculty of Agriculture and Biology, Warsaw University of Life Sciences, ul. Nowoursynowska 159/37, 02-776 Warsaw, Poland; 3 Bioremediation Department, Malopolska Centre of Biotechnology, Jagiellonian University, ul. Gronostajowa 7A, 30-387 Kraków, Poland

**Keywords:** *Anthyllis vulneraria*, Antioxidant system, Cell ultra-structure, Chl *a* fluorescence, Pb toxicity, Photosynthetic apparatus

## Abstract

Kidney vetch (*Anthyllis vulneraria* L.) is a well-known Zn hyperaccumulator. Zn often occurs with Pb in one ore; thus, plants inhabiting waste dumps are exposed not only to Zn but also to Pb toxicity. While the response of kidney vetch to Zn toxicity is relatively well known, the Pb survival strategy of *Anthyllis vulneraria* has not been the subject of investigations. The aim of presented research was to determine the survival strategy of kidney vetch exposed to high lead concentrations. Shoot explants of a calamine kidney vetch ecotype were placed on agar media containing 0.0, 0.5, 1.0, and 1.5 mM Pb. Morphological, physiological, and biochemical responses, in particular photosynthetic apparatus of plantlets, were examined. The most pronounced changes were observed in plants grown on media supplemented with 1.5 mM Pb after 8 weeks of culture. Increased dry weight and high lead accumulation were observed in roots. Similarly, in shoots, increased dry weight and a decreased number of newly formed shoots were recorded. The accumulation of lead was many times lower in shoots than in roots. In leaf cells’ ultra-structure, looser arrangement of chloroplast thylakoid grana was observed. Despite the decrease in chlorophyll *a* and carotenoid content, the photosynthetic apparatus remained efficient due to the lack of photoinhibition and increased electron transport rate beyond photosystem II (PSII). For the first time, an acclimatization mechanism based on maintaining the high efficiency of photosynthetic apparatus resulting from increasing of electron transport rate was described.

## Introduction

Despite the fact that lead emission into the environment decreased in the past few decades (mainly due to the introduction of unleaded petrol (von Storch et al. 2003)), it still has severe effects on human health (Järup 2003). There are regions where the concentration of this harmful element still is very high. These regions include waste disposal sites from mining and processing of lead ores, scattered around the world and often located near human settlements (Baker et al. 2010; Li et al. 2014; Stefanowicz et al. 2014). Wastes, deposited in the form of high heaps, are exposed to strong wind and water erosion leading to elements’ dispersion through the air (Ciarkowska et al. 2017). These processes can be limited by creating a plant cover that immobilizes heavy metals and prevents their further spread in a process referred to as phytostabilization (Mendez and Maier 2008).

Plants used in phytostabilization ought to produce biomass at the same or higher level as on uncontaminated soil and self-reproduce (Mendez and Maier 2008). Such plants must cope with the toxic effect of lead that affects physiological and biochemical processes at all levels of plant organization—from the whole organism to individual cell organelles (Sharma and Dubey 2005; Pourrut et al. 2011). Although lead is characterized by low mobility, resulting from its poor solubility (it binds to organic matter in the soil) (Kopittke et al. 2008), it can still get into the plant’s organism through adsorption on the root surface and subsequently to its interior by ionic channels (Wang et al. 2007). *In planta*, lead moves apoplastically across the root to the endodermis where it is blocked by the Casparian strip (Pourrut et al. 2011). Nevertheless, lead in high concentrations disintegrates this physical barrier (Seregin et al. 2004), enters the xylem, and translocates to the aerial parts of the plant (Verbruggen et al. 2009).

Pb exerts various adverse effects on plants. Like other non-essential heavy metals, it strongly inhibits germination and restricts plant biomass production by reducing root and shoot growth (Kopittke et al. 2007; Gopal and Rizvi 2008; Islam et al. 2008; Nagajyoti et al. 2010; Singh et al. 2010). Furthermore, lead in plant cells disturbs enzyme activity, membrane permeability, water status, mineral uptake, and photosynthesis (Sharma and Dubey 2005; Pourrut et al. 2011). Indirect Pb effect on photosynthesis results from disturbances in mineral nutrition. Its presence in the substrate limits the content of divalent cations (Zn^2+^, Mn^2+^, Ca^2+^, Fe^2+^, and Mg^2+^) in leaves (Seregin et al. 2004; Sinha et al. 2006; Gopal and Rizvi 2008; Kopittke et al. 2007). The size and spheric characteristic of lead ions closely resembles potassium; thus, it competes with K^+^ for membrane transporter binding sites. Moreover, Pb causes the efflux of K^+^ from the root by binding to K^+^-ATPase and oxidizing the SH groups of membrane proteins (Sharma and Dubey 2005). Directly, lead influences photosynthesis, affecting both the donor and the acceptor side of photosystem II (PSII). Deactivation of δ-aminolevulinic acid dehydratase (ALAD), resulting in inhibition of chlorophyll synthesis, and substituting Ca^2+^ and/or Mn^2+^ in the oxygen-evolving complex (OEC), and Mg^2+^ in the chlorophyll porphyrin ring in PSII reaction center (RC) by Pb^2+^, leads to the formation an unstable molecule in the single excitation state inactivating RC (Sharma and Dubey 2005; Harpaz-Saad et al. 2007; Romanowska et al. 2008). In addition, Pb limits the synthesis of plastoquinone, plastocyanin, and ferredoxin reductase, leading to a decrease in the electron transport rate (Romanowska et al. 2008). Moreover, it reduces synthesis of carotenoides (Kosobrukhov et al. 2004; Chen et al. 2007; Liu et al. 2008; Gupta et al. 2009; Cenkci et al. 2010). Furthermore, lead inhibits energy absorption and electron transport beyond PSII by damaging the secondary and tertiary structures of PSII proteins (Qufei and Fashui 2009). Pb impairs Calvin cycle by replacing cofactors (Mg, Fe), binding to carboxyl groups, or altering the structure of enzymes (Sharma and Dubey 2005; Gupta et al. 2009, 2010). As a result of Pb toxicity, a decrease in carboxylative but not oxidative activity of Rubisco is observed, what leads to further decrease in the efficiency of photosynthesis (Romanowska et al. 2002). Moreover, Pb disturbs photosynthesis by affecting chloroplast ultra-structure, due to its high affinity for proteins rich in amine and sulfonyl groups (Xiong et al. 2006; Hu et al. 2007; Liu et al. 2008; Piotrowska et al. 2009). Similarly as other heavy metals, Pb can cause oxidative damage (proteins, DNA, and unsaturated fatty acids oxidation) by induction of reactive oxygen species (ROS) formation. The ROS reaction with lipids, particularly with arachidonic acid which is peroxidized to finally form malondialdehyde (MDA), is generally known as “lipid peroxidation” and MDA content is widely accepted biomarker of oxidative stress (Tsikas 2017).

Plants have developed various mechanisms of coping with excess lead in the environment (Sharma and Dubey 2005; Pourrut et al. 2011; Gupta et al. 2013). Some plants utilize the avoidance strategy reducing metal uptake by secreting various organic compounds (organic acids, polysaccharides) that bind lead in the rhizosphere (Lin et al. 2004) and immobilizing Pb in cell walls by binding with pectins (Meyers et al. 2008; Krzesłowska et al. 2009; Jiang and Liu 2010; Krzesłowska 2011). Other plants have developed lead detoxification mechanisms by forming complexes with cysteine, glutathione or phytochelatins and their sequestration in vacuoles or other cytoplasmic vesicles (Wierzbicka et al. 2007; Małecka et al. 2008; Meyers et al. 2008). In addition, plants, as with other abiotic stresses, trigger a number of defensive responses in the form of increased antioxidant activity, osmolyte accumulation, or polyamine and amino acid synthesis (Michalak 2006; Qureshi et al. 2007; Gupta et al. 2010; Singh et al. 2010).

One of the species that is relatively resistant to trace elements is kidney vetch (*Anthyllis vulneraria* L.) (Turnau et al. 2010). Different *A. vulneraria* ecotypes spontaneously colonize established waste deposits or mining areas (Mahieu et al. 2011, 2013; Wójcik et al. 2014; Muszyńska et al. 2015). Moreover, *Anthyllis vulneraria* has been identified as a hyperaccumulator of zinc (Grison et al. 2015). Zinc and lead are very often deposited in the same ores, so tailings, after their extraction and processing, are rich in Zn and Pb ions, and plants growing on such wastes have to cope with the toxicity of both elements (Turnau et al. 2010; Mahieu et al. 2011, 2013; Wójcik et al. 2014; Rozpądek et al. 2018). There is a relatively large amount of data describing the response of kidney vetch to Zn toxicity (Grison et al. 2015; Mahieu et al. 2011, 2013), but the mechanisms of Pb tolerance are not known.

Therefore, the aim of this study was to determine the survival strategy of *Anthyllis vulneraria* treated with high concentrations of lead. Our goal was to evaluate the plants’ tolerance strategy in terms of the role of binding lead to cellular structures and/or the ability to adapt the photosynthetic apparatus to increased concentration of lead in the medium. To exclude other adverse factors (drought, high light intensity, pathogens, etc.) affecting plant metabolism, experiments were conducted under stable and controlled conditions of in vitro cultures.

## Materials and methods

### Plant material

A calamine ecotype of kidney vetch (*Anthyllis vulneraria* L.) from the post-mining waste dumps of Southern Poland, Boleslaw near Olkusz (Muszyńska et al. 2015), was used in the study. Seeds, collected in autumn 2012, were surfaced disinfected and placed to germinate on MS macro and microelements medium (Murashige and Skoog 1962) with 20 g L^−1^ sucrose and solidified with 10 g L^−1^ agar (Oxoid Limited, Basingstoke, Hampshire, England), pH 5.8. Shoots of the resulting seedlings were transferred and directly multiplicated on proliferation medium containing MS macro, microelements and vitamins, 500 mg L^−1^ polyvinylpyrrolidone, 650 mg L^−1^ calcium gluconate, 1.5 mg ·L^−1^ kinetin, 1.0 mg L^−1^ naphthaleneacetic acid, and 20 g L^−1^ sucrose and solidified with 8 g L^−1^ agar, pH 5.8.

### Culture conditions and stress treatment

Explants (fragments of in vitro shoots) were placed on the proliferation medium supplemented with lead nitrate (Pb(NO_3_)_2_) at concentrations 0.0, 0.5, 1.0, and 1.5 mM Pb. In vitro culture was conducted for 8 weeks. For each Pb concentration, five shoots were placed in each of eight vessels. All plants were cultured at 25 ± 1 °C under a 16 photoperiod (16 h:8 h, light:dark) and light intensity 50 μmol m^−2^ s^−1^ photosynthetic photon flux density.

### Morphological and growth parameters’ assessment

After 4 and 8 weeks of cultivation, plant growth and vitality was assessed. Shoot multiplication rate was evaluated as the number of newly regenerated shoots per explant. Average height of primary laid shoots was also measured. Additionally, rooting efficiency was estimated as rooting percentage, average number of roots per explants, and average length of the longest root per explants. Shoots and roots were separately freeze-dried for 48 h.

### Determination of lead content in plant tissues

Plant freeze-dried samples (shoots and roots after 8 weeks of culture) were subjected to open acid digestion assisted with peroxide addition. Subsequently, the digested samples were analyzed for total Pb content. This method involves very strong acid digestion that dissolves all the elements presented in the plant material (Huguet et al. 2015). Atomic absorption spectrometry (flame atomic absorption spectroscopy (FAAS) or graphite furnace atomic absorption spectroscopy (GF-AAS)), equipped with Zeeman effect background correction and an CSX 260 auto-sampler (Thermo Scientific, iC 3000), was used to determine metal concentrations. Total concentrations of Pb in plant tissues were determined after digestion of about 200 mg dry matter in 5 ml of HNO_3_ and 1.65 ml of H_2_O_2_ in an open vessel. Solutions were filtered, adjusted to 25 ml with Milli-Q® water, and analyzed.

### Determination of lipid peroxidation

The concentration of MDA, the product of lipid peroxidation, was measured by spectrophotometric method according to Dhindsa et al. (1981) in freeze-dried shoots and roots after 4 and 8 weeks of culture. Absorbance was measured at 532 and 600 nm. Absorbance at 532 nm (*A*_532_) was subtracted from the value at 600 nm (*A*_600_, the correction value): *A*_*x*_ = *A*_532_ − *A*_600_. The concentration of MDA was calculated using absorbance coefficient for MDA *ε* = 155 mM^−1^ cm^−1^.

### Phenolic compounds’ content and antioxidant enzymes’ activity determination

The photometric method with Folin’s reagent (Swain and Hillis 1959) was used to estimate phenolic compounds’ content. The absorbance of the samples was measured at 740 nm. Chlorogenic acid was used as standard. Phenolic compounds’ content was expressed as milligram of chlorogenic acid per 1 g of dry weight tissue. Additionally, content of anthocyanin was determined spectrophotometrically according to method of Fukumoto and Mazza (2000). The absorbance was measured at a wavelength 520 nm. D-cyanidin was used as standard.

Peroxidase (POD) activity was determined spectrophotometrically according to method of Lűck (1962). The absorbance of colored reaction product was measured at 485 nm, after 1 and 2 min. One unit of POD activity corresponds to an absorbance increase of 0.1.

Catalase (CAT) activity was determined spectrophotometrically according to the method described by Bartosz (2006). The absorbance of hydrogen peroxide was measured at 240 nm for 4 min in 1-min intervals. One unit of CAT activity was the amount of enzyme that decomposed 1 μmol H_2_O_2_ in 1 min. The absorbance, in all spectrophotometric methods, was measured with the use of a double-beam spectrophotometer U-2900 (Hitachi High-Technologies Corporation).

### Estimation of photosynthesis apparatus condition

#### Photosynthetic pigment content estimation

Photosynthetic pigments were determined spectrophotometrically according to Lichtenthaler (1987). Ten milligrams of freeze-dried shoot tissue were extracted with 1.0 ml of 80% acetone in ice-cold conditions. The samples were centrifuged for 15 min at 25155 g at 4 °C. After dilution, the absorbance of chlorophyll *a* (Chl *a*), chlorophyll *b* (Chl *b*), and total carotenoids (Car) was measured at 470, 646, and 663 nm, respectively, using double-beam spectrophotometer U-2900. The Wellburn (1994) equations were used to calculate the pigment content. Total chlorophylls (Chl *a+b*), the chlorophyll a/b ratio (Chl *a*/*b*), and the ratio of total carotenoids to total chlorophylls (Car/Chl *a+b*) were also calculated.

#### Chlorophyll a fluorescence measurement

After 4 and 8 weeks of cultivation, the chlorophyll *a* fluorescence induction curve analysis was performed on in vitro plant leaves. Young, fully developed, middle leaves of plant rosettes were selected for analysis (the youngest, immature, and the oldest, senescing leaves were omitted). Leaves from 12 plants of each combination were dark adapted for 25 min. Chlorophyll fluorescence induction kinetics were recorded using Handy-PEA (Hansatech, UK) spectrofluorometer according to standard procedures. The fluorescence was induced by red light: *λ*max = 650 nm, 2000 μmol (quants) m^−2^ s^−1^. Selected functional and structural photosynthetic parameters (Table 1) were calculated according to Jiang et al. (2008). Recorded curves were analyzed using the fluorometer producer’s software (PEA-Plus).

**Table 1:** Abbreviations and descriptions of extracted and calculated photosynthetic parameters (Jiang et al. 2008)

Extracted parameters	
*F*_0_	Minimum fluorescence, when all PSII reaction centers (RCs) are open
*F*_M_	Maximum fluorescence, when all PSII reaction centers are closed
*F*_50μs_, *F*_100μs_, *F*_300μs_, *F*_2ms_, *F*_30ms_	Fluorescence intensities at 50, 100, and 300 μs, and 2 and 30 ms, respectively
Area	Total complementary area between fluorescence induction curve and *F = F*_M_
Calculated parameters	
OJIP parameters	
*V*_J_	Relative variable fluorescence at 2 ms (J-step); *V*_J_ = (*F*_2ms_ − *F*_0_)/(*F*_M_ − *F*_0_)
*V*_I_	Relative variable fluorescence at 30 ms (I-step); *V*_I_ = (*F*_30ms_ − *F*_0_)/(*F*_M_ − F_*0*_)
*S*_m_	Normalized total complementary area above the OJIP transient (reflecting multiple-turnover Q_A_ reducti on events) or total electron carriers r RC; *S*_m_ = Area/(*F*_M_ − *F*_0_)
Yields or flux ratios	
*φ*_Po_	Maximum quantum yield of primary photochemistry at *t* = 0; *φ*_Po_ = 1 − *F*_0_/*F*_M_ = *F*v/*F*_M_
*φ*_Eo_	Quantum yield for electron transport at *t* = 0; *φ*_Eo_ = (*F*v/*F*_M_)(1 − *V*_J_)
*ψ*_Eo_	Probability (at time 0) that trapped exciton moves an electron into the electron transport chain beyond; *ψ*_Eo_ = 1 − *V*_J_
*ρ*_R_	Efficiency with which a trapped exciton can move an electron into the electron transport chain from Q_A_^*−*^ to the PSI and electron acceptors; *ρ*_Ro_ = *ψ*_Eo_*δ*_Ro_ = (1 − *V*_J_)(1 − *V*_I_)/(1 − *V*_J_)
*δ*_Ro_	Efficiency with which an electron can move from the reduced intersystem electron acceptors to the PSI end electron acceptors; *δ*_Ro_ = RE_o_/ET_o_ = (1 − *V*_I_)/(1 − *V*_J_)
*φ*_Ro_	Quantum yield for the reduction of end acceptors of PSI per photon absorbed; *φ*_Ro_ = RE_o_/ABS = *φ*_Po_*ψ*_Eo_*δ*_Ro_
Specific fluxes or activities per reaction center (RC)	
ABS/RC	Absorption flux per RC; ABS/RC = Mo/*V*_J_ *=* 4(*F*_300μs_ − *F*_0_)/(*F*_M_ − *F*_0_)/*V*_J_
TR_o_/RC	Trapped energy flux per RC at *t* = 0; TR_o_*/*RC *=* Mo/*V*_J_
ET_o_/RC	Electron transport flux per RC at *t* = 0; ET_o_/RC = (Mo/*V*_J_)*ψ*_Eo_
DI_o_/RC	Dissipated energy flux per RC at *t* = 0; DI_o_/RC = ABS/RC − TR_o_/RC
Phenomenological fluxes or activities per excited cross section (CS)	
ABS/CS_o_	Absorption flux per CS at *t* = 0; ABS/CS_o_ ≈ *F*_0_
TR_o_/CS_o_	Trapped energy flux per CS at *t* = 0; TR_o_/CS_o_ = (ABS/CS_o_)*φ*_Po_
ET_o_/CS_o_	Electron transport flux per CS at *t* = 0; ET_o_/CS_o_ = (ABS/CS_o_)*φ*_Eo_
DI_o_/CS_o_	Dissipated energy flux per CS at *t* = 0; DI_o_/CS_o_ = ABS CS_o_ − TR_o_/CS_o_
Density of reaction centers	
RC/CS_o_	Amount of active PSII RCs per CS at *t* = 0; RC/CS_o_ = *φ*_Po_(ABS/CS_o_)(*V*_J_/Mo)
Performance index	
PI	Performance index (PI) on absorption basis; PI = (RC/ABS)(*φ*_Po_/(1 − *φ*_Po_))(*φ*_Eo_/(1 − *φ*_Eo_))

#### TEM observation

After 8 weeks of culture, fragments of leaves were fixed in 2% paraformaldehyde and 2% glutaraldehyde in 0.1 M cacodylate buffer (pH 7.2) for 2 h, rinsed four times in cacodylate buffer, and post-fixed in a solution of 2% osmium tetroxide in cacodylate buffer for 3 h at 4 °C. The samples were dehydrated through a graded ethanol series and substituted by propylene oxide, and then embedded in glycid ether 100 epoxy resin (SERVA) equivalent to the former Epon812. The resin was polymerized at 65 °C for 24 h. Semi-thin sections were prepared with Jung RM 2065 microtome, stained with methylene blue and azur A, and examined under a light microscope (Olympus-Provis, Japan). Ultra-thin sections were prepared with Ultracut UCT Leica microtome, collected on formvar-coated grids and stained with uranyl acetate followed by lead citrate for 1 min. Examinations were made under a transmission electron microscope (Morgagni 268D).

### Statistical analyses

All results were subjected to one-way analysis of variance (ANOVA). The significant differences between means were determined using DUNCUN test at *p* < 0.05 level. Statistica 12.0 (StatSoft Inc., Tulsa, OK, USA) was used to carry out statistical analyses. Results for growth parameters were obtained from 20 replicates. Results for dry weight content, MDA content, phenolic compound content, pigment content, and enzyme activities were obtained from 5 replicates. Results for Pb content were obtained from 3 replicates and for the chlorophyll *a*, fluorescence induction curves from 12 replicates. Results obtained for the shoots and roots from 4- and 8-weeks in vitro plants were separately statistically verified.

## Results

### Growth and development of explants on lead-containing media

**Fig. 1:**
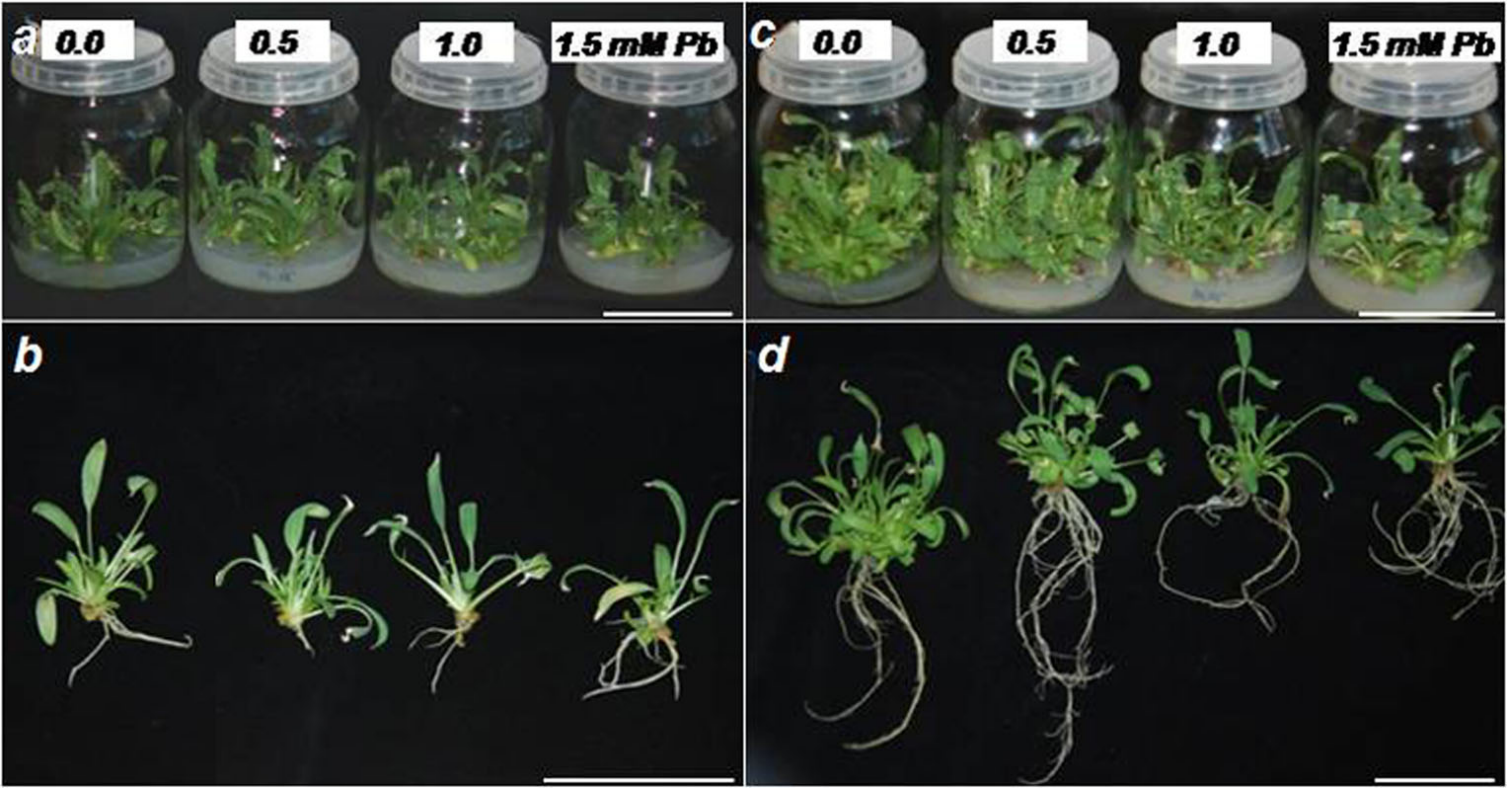
Kidney vetch in vitro plants after 4 (**a**, **b**) and 8 (**c**, **d**) weeks of cultivation on media with different Pb concentration. Scale bars, 5 cm

After 4 weeks, no statistically significant changes were observed in the shoot multiplication rate of kidney vetch on media containing different concentrations of Pb (Table 2, Fig. 1a, b). The shoot multiplication rate averaged from 2.7 to 4.3 shoots/explant (Table 2). After 8 weeks, a significant decrease in shoot regeneration was observed on media containing the highest Pb concentration (Table 2, Fig. 1c, d). No statistically significant differences were shown in plants’ height. During cultivation, shoots (explants) spontaneously formed roots. However, no significant differences in rooting percentage of shoots were observed regardless of the Pb concentration in the media (Table 2). No significant differences in the average number of roots produced by a single shoot were shown; however, after 8 weeks of culture, a decrease in the average root number was shown in the case of shoots cultivated on the medium with the highest concentration of lead ions (Table 2). Cultivation under 1.5 mM of Pb induced a significant decline in root length (2.6 cm compared to 4.0 cm in control) after 4 weeks (Table 2). On the other hand, after 8 weeks of culture, on media containing 0.5 mM Pb, longer roots (10.7 cm compared to 7.3 cm) were produced (Table 2).

**Table 2:** Shoot multiplication rate, average shoot height, rooting efficiency, and dry weight content of kidney vetch depending on Pb concentration in the medium after 4 and 8 weeks of culture

Parameter^a^		Period	Pb [mM]			
			0.0	0.5	1.0	1.5
Shoot multiplication rate ± SD		4 weeks	3.5 ± 1.1^ab*^	4.3 ± 1.5^a^	3.9 ± 1.8^a^	2.7 ± 0.8^b^
		8 weeks	6.5 ± 2.5^ab^	7.0 ± 2.0^a^	5.3 ± 2.0^b^	4.0 ± 1.4^c^
Average shoot height (cm) ± SD		4 weeks	5.0 ± 1.1^a^	5.1 ± 0.7^a^	5.3 ± 1.0^a^	4.7 ± 0.6^a^
		8 weeks	6.0 ± 1.3^a^	5.7 ± 1.3^a^	5.9 ± 1.3^a^	5.3 ± 1.0^a^
Rooting efficiency	Rooting (%) ± SD	4 weeks	60.0 ± 16.3^a^	80.0 ± 16.3^a^	60.0 ± 28.3^a^	75.0 ± 19.1^a^
		8 weeks	85.0 ± 19.1^a^	75.0 ± 19.1^a^	80.0 ± 16.3^a^	75.0 ± 30.0^a^
	Average root number/explants ± SD	4 weeks	9.2 ± 4.8^a^	6.6 ± 4.6^a^	8.0 ± 5.2^a^	6.0 ± 5.1^a^
		8 weeks	9.8 ± 5.8^ab^	9.9 ± 4.3^ab^	11.6 ± 4.6^a^	6.7 ± 4.0^b^
	Average root length (cm) ± SD	4 weeks	4.0 ± 0.9^a^	3.9 ± 1.7^a^	3.6 ± 1.3^ab^	2.6 ± 1.2^b^
		8 weeks	7.3 ± 3.6^b^	10.7 ± 3.6^a^	8.9 ± 3.1^ab^	8.6 ± 3.1^ab^
Percentage of dry weight content (%) ± SD	Shoot	4 weeks	10.4 ± 1.1^a^	11.6 ± 1.8^a^	10.9 ± 0.6^a^	10.8 ± 1.1^a^
		8 weeks	8.4 ± 0.7^b^	7.9 ± 0.4^b^	8.4 ± 0.7^b^	10.4 ± 0.8^a^
	Root	4 weeks	7.4 ± 0.5^b^	9.5 ± 1.1^a^	8.6 ± 1.1^a^	9.7 ± 0.5^a^
		8 weeks	8.9 ± 1.5^b^	9.4 ± 0.5^b^	9.5 ± 1.2^b^	12.3 ± 1.0^a^

^a^*n* = 20; rooting percentage *n* = 4; dry weight content *n* = 5

*Different letters—statistically significant difference within each period and organ at *p* ≤ 0.05

After 4 weeks of culture, the percentage of dry weight did not change in shoots but increased in roots on media containing Pb ions (Table 2). Interestingly, after 8 weeks of culture, a statistically significant increase in percentage of dry weight was recorded both in the shoots and in the roots of plants cultivated on medium supplemented with 1.5 mM Pb (Table 2).

### Lead content in leaves and roots of kidney vetch

**Fig. 2:**
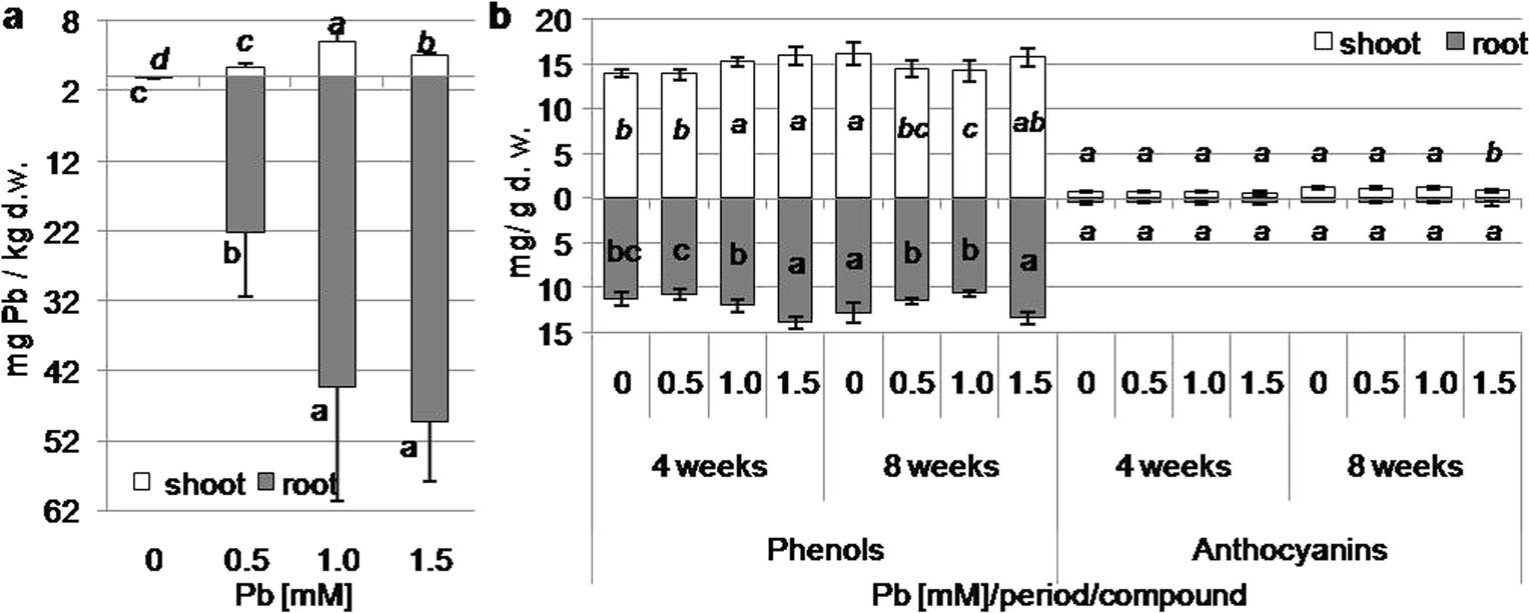
Lead (**a**) and phenolic compound (**b**) content in shoots and roots of kidney vetch depending on Pb concentration in the medium after 8 weeks (**a**) and 4 and 8 weeks (**b**) of culture (*different letters*—statistically significant difference within each organ, period and compound at *p* ≤ 0.05, *n* = 3 (lead content) and 5 (phenolic compound content))

The lead content in leaves of plants cultivated on lead-containing media ranged from 1.34 to 5.01 mg/kg d.w. (Fig. 2a). The highest accumulation of lead was observed in mature leaves of plants cultivated on medium with 1.0 mM Pb (Fig. 2a). In turn, increasing the concentration of lead in the medium to 1.0 mM Pb resulted in a statistically significant increase in the accumulation of Pb in the roots (Fig. 2a).

### Plasma membrane integrity of kidney vetch shoots and roots on lead-containing media

**Fig. 3:**
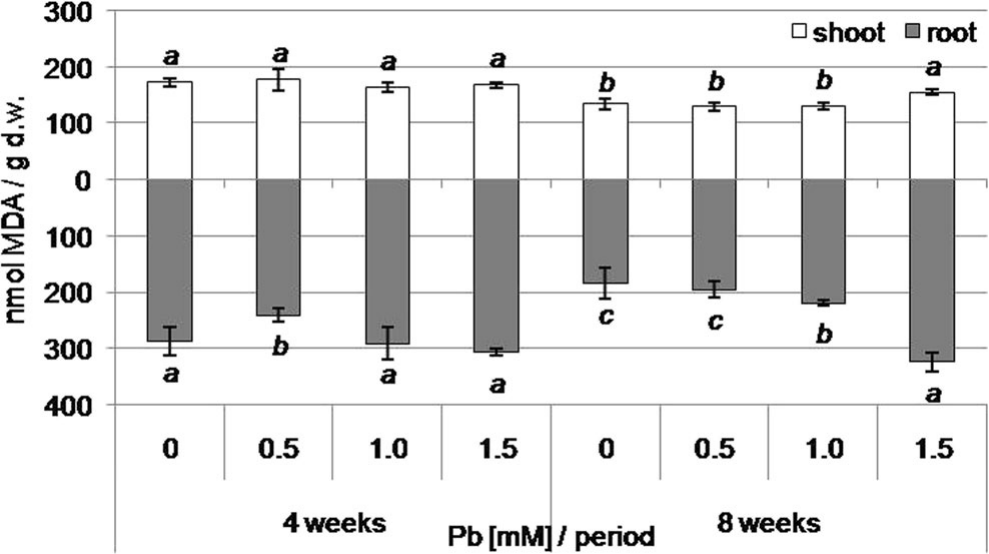
Malondialdehyde (MDA) content in shoots and roots of kidney vetch depending on Pb concentration in the medium after 4 and 8 weeks of culture (*different letters*—statistically significant difference within each period and organ at *p* ≤ 0.05, *n* = 5)

The increase in the content of MDA indicates increased lipid peroxidation and cell membrane permeability. In regenerated shoots, an increase in the MDA concentration, in comparison to control shoots, was observed only after 8 weeks of cultivation in plants from media containing 1.5 mM Pb (Fig. 3). In turn, MDA content decreased in the roots formed on medium supplemented with 0.5 mM Pb after 4 weeks of culture. MDA content increased in roots from media containing 1.0 and 1.5 mM Pb after 8 weeks (Fig. 3). The highest concentration was shown in the roots from media containing the highest Pb concentration (Fig. 3).

### Antioxidant activity of kidney vetch shoots and roots on lead-containing media

**Fig. 4:**
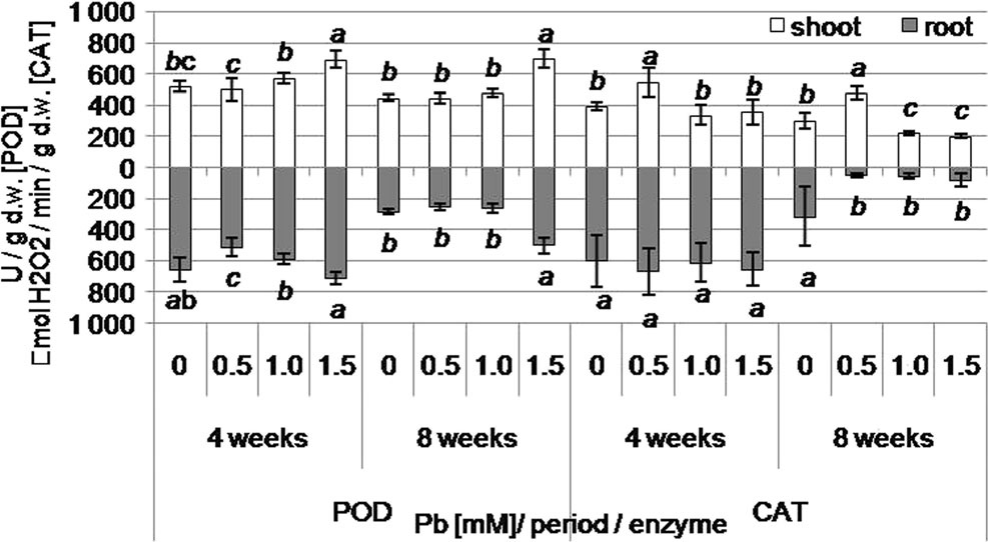
Peroxidase (POD) and catalase (CAT) activity in shoots and roots of kidney vetch depending on Pb concentration in the medium after 4 and 8 weeks of culture (*different letters*—statistically significant difference within each enzyme, period, and organ at *p* ≤ 0.05, *n* = 5)

POD activity increased, both after 4 and 8 weeks, in shoots regenerated on media containing 1.5 mM Pb (Fig. 4). In turn, POD activity decreased in roots from medium with 0.5 mM Pb after 4 weeks but after 8 weeks increased in roots from medium supplemented with 1.5 mM Pb (Fig. 4). Catalase activity, after 4 and 8 weeks, increased in shoots cultivated on medium with 0.5 mM Pb, while after 8 weeks, decreased in shoots from media containing 1.0 and 1.5 mM Pb (Fig. 4). In roots, CAT activity did not change after 4 weeks, but after 8 weeks it decreased in roots from lead-containing media (0.5–1.5 mM) (Fig. 4).

After 4 weeks of cultivation, the concentration of phenolic compounds increased in shoots regenerated on media with 1.0 and 1.5 mM Pb. After 8 weeks, the accumulation of phenols was reduced in shoots from 0.5 and 1.0 mM Pb-containing media (Fig. 2b). After 4 weeks, the phenolic content increased in the roots from the medium with the highest Pb concentration, and after 8 weeks, it decreased in roots from the medium with 0.5 and 1.0 mM Pb (Fig. 2b). The anthocyanin content was reduced only in regenerated shoots on medium with 1.5 mM Pb after 8 weeks (Fig. 2b). However, the anthocyanin concentration was not altered in roots under lead treatment (Fig. 2b).

### Photosynthetic apparatus of kidney vetch under lead stress

#### Photosynthetic pigments’ concentration

After 4 weeks of culture, the photosynthetic pigment concentration increased in shoots regenerated on medium supplemented with higher concentrations of Pb (1.0 and 1.5 mM) (Table 3). Furthermore, chl *a*/*b* ratio increased in shoots from medium with 0.5 mM Pb (Table 3). After 8 weeks of culture, a decrease in chlorophyll *a* and carotenoid content was observed in shoots growing on 1.5 mM Pb concentration media (Table 3). In contrast, the chl *a/b* ratio increased in shoots cultivated on medium with 0.5 mM Pb and decreased in shoots from medium with 1.5 mM Pb (Table 3). The ratio of carotenoids to chlorophyll *a+b* (car/chl *a+b*) increased in plants from higher concentrations of Pb after 4 weeks and decreased after 8 weeks (Table 3).

**Table 3:** Photosynthetic pigments’ content and ratios of kidney vetch depending on Pb concentration in the medium after 4 and 8 weeks of culture

Parameters^a^ ± SD	Period	Pb [mM]			
		0	0.5	1	1.5
Chlorophyll *a* (mg/g d.w.)	4 weeks	3.57 ± 0.10^c*^	3.62 ± 0.22^bc^	3.93 ± 0.19^ab^	3.93 ± 0.38^a^
	8 weeks	3.83 ± 0.12^a^	3.89 ± 0.25^a^	3.97 ± 0.26^a^	3.44 ± 0.40^b^
Chlorophyll *b* (mg/g d.w.)	4 weeks	0.98 ± 0.04^b^	0.98 ± 0.051^b^	1.08 ± 0.04^a^	1.11 ± 0.10^a^
	8 weeks	1.11 ± 0.06^a^	1.10 ± 0.07^a^	1.14 ± 0.08^a^	1.02 ± 0.12^a^
Chlorophyll *a+b* (mg/g d.w.)	4 weeks	4.55 ± .21^c^	4.60 ± 0.27^bc^	5.00 ± 0.23^ab^	5.11 ± 0.47^a^
	8 weeks	4.94 ± 0.18^a^	4.99 ± 0.32^a^	5.11 ± 0.34^a^	4.46 ± 0.51^a^
Carotenoids (mg/g d.w.)	4 weeks	0.81 ± 0.04^c^	0.83 ± 0.06^bc^	0.91 ± 0.05^ab^	0.93 ± 0.08^a^
	8 weeks	0.97 ± 0.02^a^	0.96 ± 0.06^a^	0.95 ± 0.06^a^	0.84 ± 0.09^b^
Chl *a*/Chl *b*	4 weeks	3.63 ± 0.03^b^	3.69 ± 0.06^a^	3.65 ± 0.05^ab^	3.60 ± 0.02^b^
	8 weeks	3.46 ± 0.09^b^	3.54 ± 0.03^a^	3.49 ± 0.02^ab^	3.38 ± 0.03^c^
Car/Chl *a+b*	4 weeks	0.179 ± 0.001^b^	0.180 ± 0.002^ab^	0.181 ± 0.002^a^	0.182 ± 0.002^a^
	8 weeks	0.196 ± 0.004^a^	0.193 ± 0.001^b^	0.186 ± 0.002^d^	0.189 ± 0.002^c^

^a^*n* = 5

*Different letters—statistically significant difference within each parameter and period at *p* ≤ 0.05

#### Chlorophyll a fluorescence of kidney vetch on lead-containing media

**Fig. 5:**
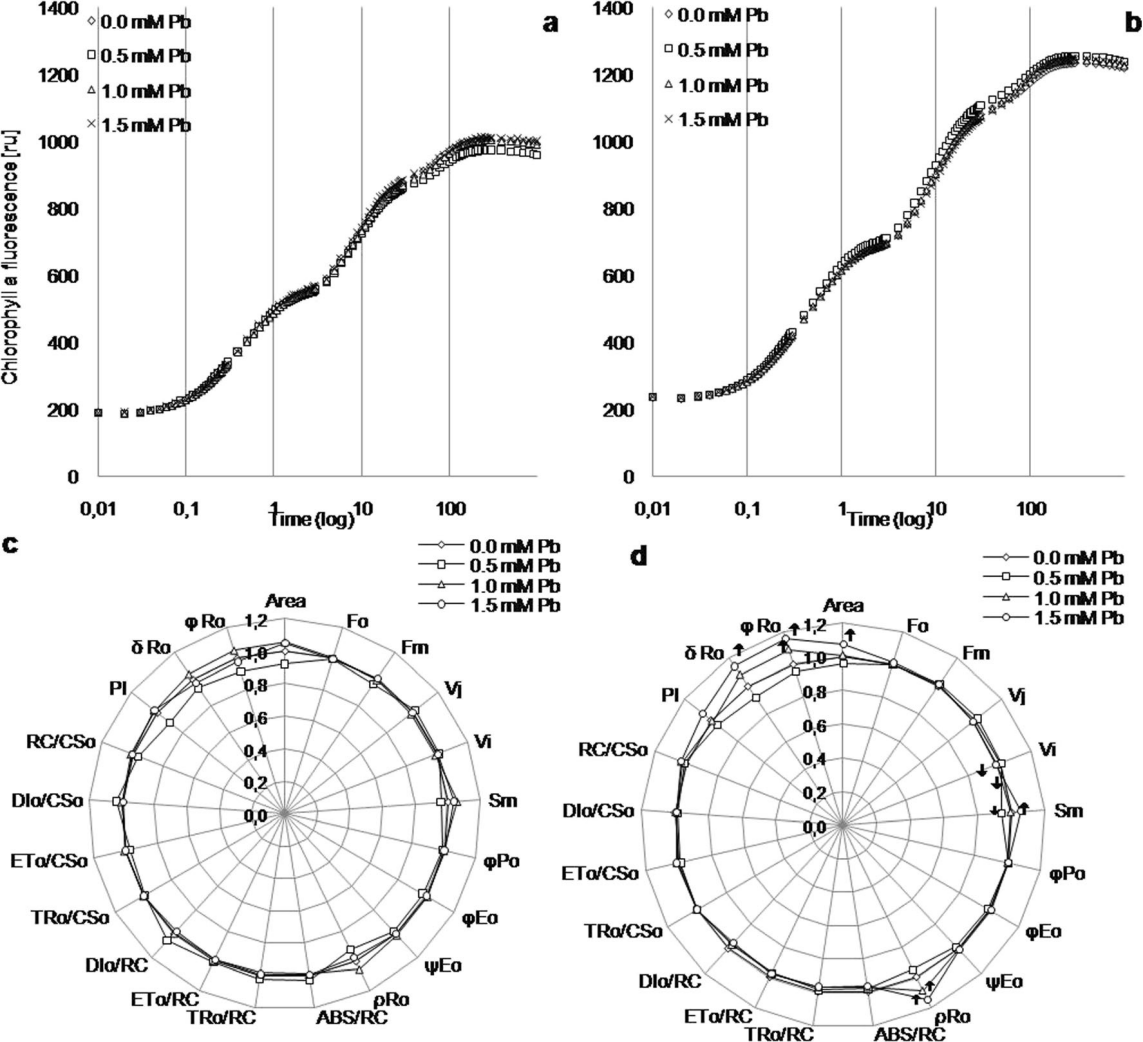
The fast chlorophyll *a* fluorescence transient (**a**, **b**) and structural and functional parameters of photosystem II (**c**, **d**) of kidney vetch leaves depending on Pb concentration in the medium after 4 (**a**, **c**) and 8 (**b**, **d**) weeks of culture. ru, relative units; all the values in **c** and **d** were expressed relative to the control (set as 1); abbreviations—see Table 1; up and down arrows, statistically significant increase or decrease within each parameter in relation to control at *p* ≤ 0.05, *n* = 12

The fast chlorophyll *a* fluorescence transient of kidney vetch leaves had a typical OJIP rise (Fig. 5a, b). All of the measured PSII parameters are presented on the radar chart (Fig. 5c, d). The values were normalized against the control value. After 4 weeks of culture, different Pb concentrations in media solution had no significant impact on O, J, I, and P transients indicating no effect of lead on the photosynthetic apparatus (Fig. 5a, c). In turn, after 8 weeks, decreased values of *V*_I_ in leaves on 1.0 and 1.5 mM Pb and *S*_m_ in leaves on 0.5 mM Pb as well as area, *S*_m_, *ρ*_Ro_, *φ*_Ro_, and *δ*_Ro_ values increase in 1.5 mM Pb medium compared to control leaves were detected (Fig. 5d).

#### Chloroplast ultra-structure

**Fig. 6:**
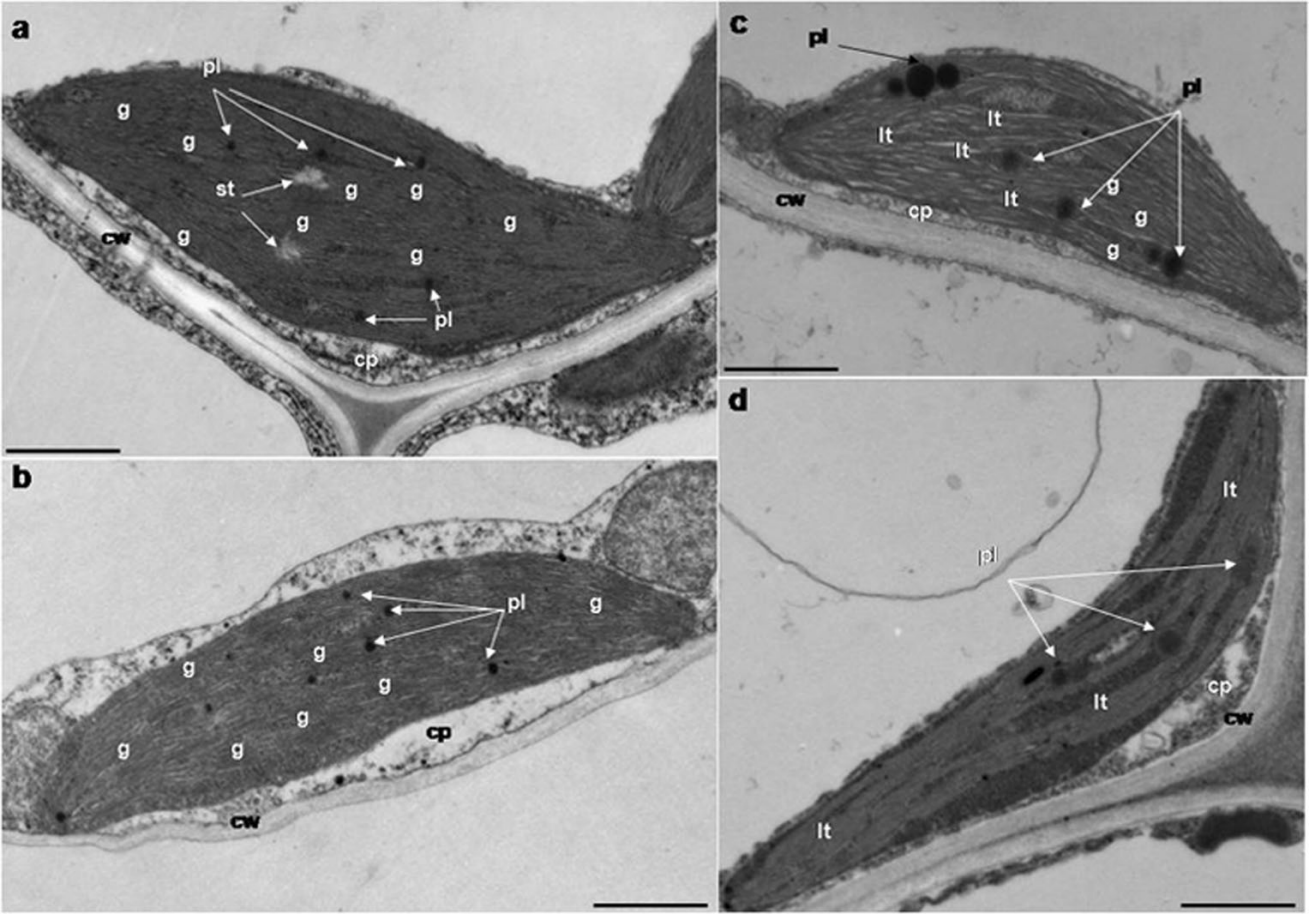
Lead (Pb) effects on the ultra-structure of kidney vetch chloroplasts. Chloroplasts of plants from media containing 0.0 mM Pb (**a**), 0.5 mM Pb (**b**), 1.0 mM Pb (**c**), and 1.5 mM Pb (**d**). cw, cell wall; cp, cytoplasm; st, starch; g, grana; pl, plastoglobule; lt, loose thylakoids. Scale bars, 1 μm

Cell ultra-structure analysis revealed differences between chloroplasts from leaves from media with different Pb concentrations (Fig. 6). Chloroplasts of plants from 0.0 and 0.5 mM Pb media had regular structure, a compact arrangement of thylakoids, numerous grana, a few small plastoglobules, and sometimes starch grains (Fig. 6a, b). Generally, in cells of leaves from plants cultivated on lead supplemented media fewer chloroplasts were observed. In the leaves of plants from 1.0 and 1.5 mM Pb medium, among normally formed chloroplasts, smaller ones appeared, with a looser arrangement of thylakoids and grana and containing larger plastoglobules (Fig. 6c, d).

## Discussion

Lead, naturally occurring in soils, originates from the parent rock (Pourrut et al. 2011). According to various reports, the average lead concentration in unpolluted soils ranges from 17 mg kg^−1^ (Alloway 2013), 25 mg kg^−1^ (Kabata-Pendias and Mukherjee 2007), up to almost 50 mg kg^−1^ (Pais and Jones 2000). It is assumed that lead concentration in agricultural soils above 100 mg kg^−1^ is phytotoxic (Pais and Jones 2000). In turn, the allowable Pb concentration for industrial area soils can reach 600 mg kg^−1^ (Wójcik et al. 2014). Total lead concentrations in extremely contaminated soils reach up to 7000 mg kg^−1^ along high traffic roads (Kabata-Pendias and Mukherjee 2007) and up to 25,300 mg kg^−1^ in Pb ore tailings (Mahieu et al. 2011). Total Pb concentration does not relate to its mobility and accessibility for plants. Wójcik et al. (2014) estimated that only 0.005–0.03% of total Pb deposited in soils is extractable and mobile. It is due to association of Pb with soil organic matter hydroxides (Kabata-Pendias and Mukherjee 2007). Interestingly, the highest amount of accessible lead was found in samples taken from the oldest Zn–Pb ore waste heap that was covered by plants including *Anthyllis vulneraria.* The Pb concentrations used in this study were selected according to previous *experiments* (Muszyńska et al. 2017) and ranged between 100 and 300 mg L^−1^ of total Pb.

A visible symptom of the toxic effects of lead on plants is disturbance of growth and development (Sharma and Dubey 2005; Pourrut et al. 2011). Interference in the intake of essential nutrients or photosynthesis disorders induced by lead ions may cause organ shortening and plant biomass reduction (Lamhamdi et al. 2013; Wang et al. 2016). Growth disorders are caused by disturbances on the cellular level (Sharma and Dubey 2005; Gopal and Rizvi 2008). Lead ions cause abnormality in the cytoskeleton, leading to impairment of the uptake and distribution of organic and mineral compounds (Sharma and Dubey 2005; Gopal and Rizvi 2008) and the formation of cario- and cytokinetic spindles limiting cell divisions (Patra et al. 2004). Many authors report that the presence of lead caused a rapid reduction in root growth (Islam et al. 2007; Kopittke et al. 2007; Liu et al. 2008). In presented studies, only at the highest Pb concentration applied, after 4 weeks of cultivation, the reduction of root growth was observed in kidney vetch plants. Moreover, the Pb retardation effect on the roots has disappeared after another 4 weeks of culture. Many authors observed also limitations in aerial plant parts’ growth in various plant species exposed to lead (Gopal and Rizvi 2008; Gupta et al. 2009; Piotrowska et al. 2009; Singh et al. 2010). Contrary to literature data, our study revealed that *Anthyllis vulneraria* shoot elongation was not inhibited regardless of Pb concentration used. Solely, the decrease in the number of newly formed shoots on the medium with 1.5 mM Pb after 8 weeks of culture was recorded. It appears that in *Anthyllis vulneraria* plants Pb did not affect growth and development rate when cultivated in medium containing 0.5 and 1.0 mM Pb, probably due to efficient detoxification mechanism.

The basic mechanism of avoiding the negative effect of Pb is its immobilization in cell walls by pectins (Meyers et al. 2008; Jiang and Liu 2010). Binding of Pb with pectin carboxyl groups increases cell wall thickness (dry weight increase) and is a physical barrier protecting the plasma membrane against Pb toxicity (Krzesłowska et al. 2009). In addition to binding to polysaccharides, lead can also be bound by the phenolic compounds used in cell wall lignification (Passardi et al. 2004; Michalak 2006). The root ultra-structure study of plants subjected to high lead concentrations reveals its high accumulation in the cell walls, vacuoles, and intercellular spaces (Almeida et al. 2007; Pourrut et al. 2013) and small amounts in other organelles (Sharma and Dubey 2005; Austin et al. 2006). In our study, an increase in the Pb content in the root dry weight after 8 weeks of culture at higher lead concentrations came out probably of such accumulation. It appears that the dry weight increase, as the lead effect, resulted from an efficient detoxification mechanism not directly combined with phenols since the phenolic compound content in kidney vetch plants increased after 4 weeks of cultivation and decreased after 8 weeks.

Phenol compounds, in addition to cell wall lignification, are responsible for scavenging reactive oxygen species (ROS). The formation of ROS is related to oxidative stress caused by environmental factors including heavy metals (Liu et al. 2008; Pourrut et al. 2008; Grover et al. 2010; Yadav 2010). Apart from phenols, the enzymatic antioxidant system, including catalase and peroxidase, is involved in scavenging excess ROS (Qureshi et al. 2007; Gupta et al. 2010; Singh et al. 2010). Wang et al. (2016) reported that catalase and peroxidase activity of *Dimocarpus longan* decreased with increasing Pb concentration. Unlike the previous study, our experiments with *Anthyllis vulneraria* indicated POD activity with parallel Pb concentration increase, indicating free radical scavenging and protection against oxidative damage. In turn, *Anthyllis vulneraria* plants cultivated at the lowest Pb concentration exhibited an increase but at higher Pb content, decrease in CAT activity.

In addition to ROS generation, lead causes changes in membrane lipid composition (increased level of unsaturated fatty acids vs saturated) as well as potassium leakage and lipid peroxidation (Singh et al. 2010). Experiments conducted by Shakoor et al. (2014), Kanwal et al. (2014), and Wang et al. (2016) demonstrated lipid peroxidation increase as a result of Pb treatment. In our experiment, only after 8 weeks of culture, in 1.0 and 1.5 mM Pb, an increase of lipid peroxidation in *Anthyllis vulneraria* roots was observed. This proves that the mechanisms of lead immobilization in the cell wall or intercellular spaces in *Anthyllis vulneraria* roots were sufficient in a short period, independently of Pb concentration and not efficient enough in the long term and high Pb content.

Most plant species (~ 95%) accumulate lead, acquired from the substrate, in roots and only a few have the ability to translocate Pb to aerial parts (Pourrut et al. 2011). Transport of Pb from roots to shoots is controlled by root endoderm, which is the physical barrier in apoplastic transport. In the cell walls of endoderm, lead is sequestrated or removed from the plant (Pourrut et al. 2011). However, at high Pb concentrations, endoderm cells are damaged and lead moves to the xylem (Seregin et al. 2004). In shoot cells, Pb can be chelated in the cytoplasm or vacuole (Tong et al. 2004). Moreover, Reis et al. (2015) in *Theobroma cacao* treated with 0.8 g L^−1^ Pb described the deleterious effect of Pb on lipid membrane and photosynthetic apparatus. Contrary, in *Anthyllis vulneraria* leaves, despite the increase in lipid peroxidation, plants cultivated on 1.5 mM Pb restricted transport to aerial parts more effectively, thus, protecting the photosynthetic apparatus.

In leaves, Pb severely impacts the photosynthetic apparatus (Sharma and Dubey 2005; Pourrut et al. 2011; Dao and Beardall 2016). The main sites of Pb action on the photosynthetic apparatus are (1) on the donor side of PSII—the OEC, in which lead causes an inhibition of this complex (Kalaji et al. 2016). (2) On the PSII RC—substitution of Mg^2+^ by Pb^2+^ in the chlorophyll porphyrin ring, what is very dangerous especially in PSII RCs and antenna systems (Harpaz-Saad et al. 2007). (3) On the acceptor side—substitution Fe^2+^ by Pb^2+^ in protonation complex which is responsible for the protonation of the semichinol radical Q_B_^−^ (Belatik et al. 2013). Since chl *a* fluorescence measurement is a useful, non-invasive, and reliable method for estimation of heavy metal impact on photosynthetic apparatus (Papageorgiou et al. 2007; Kodru et al. 2015; Stirbet et al. 2014; Kalaji et al. 2016), the fast fluorescence chl *a* kinetics test was used to assess the condition of *Anthyllis vulneraria* photosynthetic apparatus. In dark adapted leaves, all PSII RCs, Q_A_ as well as Q_B_, are fully oxidized what allows to determinate minimum fluorescence (*F*_0_), whereas maximum fluorescence (*F*_M_) is estimated after saturated light applying when all RCs and Q_A_ and Q_B_ are closed (Strasser et al. 2004). Between these two extreme points, a fast multiphasic rise in microsecond to second range–O-J-I-P transient could be measured what allows to investigate several photosynthetic events (Kodru et al. 2015). The rise of *F*_0_ is caused by limitation on the donor side due to perturbances in OEC efficiency leading to increase non-reduced PSII RCs pool in the pool of all PSII RCs (Strasser et al. 2004; Stirbet et al. 2014). An increase of *F*_0_ was reported by many authors as an effect of heavy metal intoxication. According to Sigfridsson et al. (2004), Cd^2+^ affected PSII RC causing increase of *F*_0_ value. Jiang et al. (2008) in *Citrus grandis* leaves reported *F*_0_ increase induced by Al presence. Similarly, Zhou et al. (2017) noted *F*_0_ rise in *Robinia pseudoacacia* leaves exposed to high Pb concentration due to loss of PSII RCs and their inactivation. Our results revealed that neither duration of stress treatment nor Pb concentration influenced the *F*_0_, as well as *F*_M_ values of *Anthyllis vulneraria* leaves, which was not observed by other authors, previously.

Belatik et al. (2013) reported that Pb^2+^ disrupted the e^−^ transport between Q_A_^−^ and Q_B_, leading to the inactivation of PSII on the acceptor side. These disorders were manifested by a sharp increase in the rate of I-step emergence and a decrease in its value, which was observed in the presence of both Al (Jiang et al. 2008) and Pb (Kalaji and Loboda 2007; Dao and Beardall 2016; Kalaji et al. 2016). Moreover, PSII inactivation on the acceptor side is related to e^−^ transport rate beyond Q_B_ via PSI to ferredoxin (Fd) (Stirbet et al. 2014). PSI is relatively resistant to Pb^2+^, but this metal impacts the physico-chemical properties of membranes, affecting e^−^ transport by membrane transporters (PQ pool, PC, and Fd) (Dao and Beardall 2016). Disorders at this stage are manifested by both *F*_M_ value decrease and faster and less slope course of the I–P phase of transient fluorescence (Stirbet et al. 2014). It is considered that the I–P transient increase correlates with CO_2_ assimilation rate (Schansker et al. 2005; Jiang et al. 2008). So the perturbances in this phase indicate a serious, negative impact on photosynthetic apparatus (Kalaji and Loboda 2007; Zurek et al. 2014; Dao and Beardall 2016; Kalaji et al. 2016). Also, decrease of *δ*_Ro_, *φ*_Ro_, and *ρ*_Ro_ parameters confirms that observation (Jiang et al. 2008). Our results revealed that in the case of *Anthyllis vulneraria* plants, after 8 weeks of culture regardless of Pb concentration, no reduction in the amount of energy absorbed per RC (ABS/RC), number of active reaction centers (RC/CS), and increase of energy dissipation (Di/CS) was observed, indicating low photoinhibition risk. Moreover, intermediate and the highest concentrations of Pb (1.0 and 1.5 mM) resulted in a significant increase in *Q*_B_ oxidation (*δ*_Ro_), the efficiency of quantum yield for reduction of PSI end acceptors (*φ*_Ro_), and the efficiency of electron transport (*ρ*_Ro_), especially from *Q*_B_ to the PSI end electron acceptors (*V*_I_ decrease). It is noteworthy that both donor and acceptor sides as well as the PSII RCs in *Anthyllis vulneraria* plants were not affected by Pb which was not observed previously by other authors. We hypothesize that more efficient electron transport resulted from a greater amount of end acceptors of the PSI acceptor side. Additionally, in the highest Pb concentration, faster electron transport rate was due to the higher amount of total electron carriers per RC (Sm) which was mainly due to the larger PQ pool (area). Such results were not observed by any authors previously.

Linear electron transport (LEF) between PSII RC and PSI end acceptors is located in the compressed areas of the thylakoids and on the boundary of the grana and stroma. On the other hand, cyclical e^−^ transport (CEF) refers to stromal thylakoids (Bukhov and Carpentier 2004; Joliot and Joliot 2006; Garstka 2007). The three-dimensional structure of the thylakoid membranes with the division into granal and stromal thylakoids is stabilized by the presence of cations, especially Mg^2+^. Formation of the granular system is associated with a specific membrane composition in which the dominant protein is LHCII_3_ and the lipid component is monogalactosyl diacylglycerol (MGDG) (Garstka 2007). The LHCII_3_ proteins, through the van der Waals effects, combine to form microdomains in the thylakoid membranes, leading to their coiling and spontaneous granum formation. Additionally, the structure of the forming grana is maintained by large, spatial proteins occurring in stroma, e.g., RUBISCO (Dekker and Boekema 2005; Kim et al. 2005; Garstka 2007). The PSII complexes, especially PSIIα, dominate in the compressed areas of the thylakoids, whereas PSI in the non-compressed (Dekker et al. 2002; Kirchhoff et al. 2004). The unstacking process is associated with disorders in the stabilization of the membrane system and is related to the exchange of Mg^2+^ to Pb^2+^, as well as a decrease in cation or/and increase in anion concentration (Jajoo et al. 2001). During this process, PSIIα is degraded to PSIIβ and LHCII_3_ and PSII and PSI is randomly distributed (Garab and Mustárdy 2000; Dekker et al. 2002; Kirchhoff et al. 2004). The diameter of the grana increase and some of them are transformed into stromal thylakoids (Kaftan et al. 2002). The destabilization of the chloroplast structure under the influence of heavy metals has been observed by other researchers (Reis et al. 2015; Zhou et al. 2017). Zhou with co-workers (2017) observed swollen chloroplasts without starch grains. Moreover, they reported the disappearance of the grana structure and the rapid decline of the number of thylakoids, whose arrangement became very loose. In the chloroplasts of leaves from plants treated with the highest Pb ion concentration (1 g kg^−1^), they observed total degradation of thylakoids and the outflow of lipid droplets from degraded membranes. It is worth emphasizing that in chloroplasts of kidney vetch, no significant changes in the internal organization were observed with respect to control regardless of the Pb concentration. Only in chloroplasts from plants cultivated on media containing 1.0 and 1.5 mM Pb after 8 weeks larger plastoglobules appeared, what indicated a mild effect of lead on membrane structures. On the other hand, a slightly looser arrangement of both granal and stromal thylakoids could have resulted from the reduced amount of carotenoids and chlorophyll *a*. Chlorophyll *a* is a crucial molecule since it stated at least 50% of LHCII co-participated in the spontaneous formation and stabilization of the grana. The presence of plastoglobules may be related to their function in the synthesis, repair or removal of metabolites during environmental and ontogenetic changes, and contribution to the rebuilding of membrane lipids under stress (Rottet et al. 2015). These structures not only serve as a reservoir of neutral lipids but primarily participate in their synthesis (Ytterberg et al. 2006; Vidi et al. 2006; Lundquist et al. 2012). Reducing chl *a* content leading in looser arrangement of thylakoids requires an increase in the proportion of neutral lipids for spatial stabilization of the photosynthetic apparatus. We conclude that enlargement of plastoglobules, in *Anthyllis vulneraria* chloroplasts, may be evidence of increased synthesis of neutral lipids to avoid possible dysfunction of the photosynthetic apparatus. This however, requires further examinations.

## Conclusion

In this study, the response of *Anthyllis vulneraria* to Pb was described and linked with potential mechanism/strategy to cope with lead toxicity for the first time. Binding of Pb in root cells and slight changes in the rate of growth and development, as well as the efficiency of the photosynthetic apparatus of *Anthyllis vulneraria* plants grown at high lead concentrations, indicate an acclimatization mechanism not observed by other authors. This mechanism is composed of two elements: one is increased synthesis of metabolites (higher dry weight), involved in lead inactivation, by reduction development of aerial plants’ part. The second element is the carotenoids and chl *a* content decrease, under lead stress, combined with an increase of the linear electron transport efficiency beyond PSII as well as the plastoquinone pool, indicating the acclimatization of the photosynthetic apparatus. This allows for the most efficient use of light radiation without exposing the plant organism to photooxidation.

Nevertheless, to verify *Anthyllis vulneraria* Pb acclimatization mechanism described herein, it is necessary to extend studies to natural conditions (the post-mining waste dumps that are currently in progress). However, the verification of the hypotheses about plant acclimatization to particular heavy metal will be very difficult due to many uncontrolled conditions occurring during field tests.
